# Shelf-life in cucurbitacin-containing phytonematicides: Non-conformity to Arrhenius model

**DOI:** 10.1371/journal.pone.0227959

**Published:** 2020-02-12

**Authors:** Phatu W. Mashela, Ebrahim Shokoohi, Kgabo M. Pofu

**Affiliations:** 1 University of Limpopo, Green Biotechnologies Research Centre of Excellence, Sovenga, Republic of South Africa; 2 Agricultural Research Council-VOP, Pretoria, Republic of South Africa; ICAR- Indian Agricultural research Institute, INDIA

## Abstract

Shelf-life information provides end-users with the assurance that the product is still in compliance with label claims. Behavioral reaction orders of the Arrhenius model had been consistently used under fixed conditions to provide shelf-life in food products. Due to non-conformity of the cucurbitacin-containing phytonematicides to the Arrhenius behavioral reaction orders, an alternative quadratic model consistent with the behavioral reaction orders of cucurbitacins was developed under chilled (5°C at 95–98% RH) and fixed tropical (38°C at 90% RH) conditions, while room temperature constituted unfixed conditions. Sampling for cucurbitacins was done at time-frames compliant with geometric series, with cucurbitacin analysis regularly performed using high-performance liquid chromatography techniques. Under chilled conditions, neither the Arrhenius nor the quadratic model could predict the shelf-life for Nemarioc-AL phytonematicide, whereas Nemafric-BL phytonematicide had shelf-life of 35 weeks. In contrast, under tropical conditions, the positive quadratic models showed that Nemarioc-AL and Nemafric-BL phytonematicides had shelf-life of 35 and 825 weeks, respectively. In conclusion, the two phytonematicides could be stored under fixed tropical conditions to enhance the shelf-life of their active ingredients.

## Introduction

**Fig 1:**
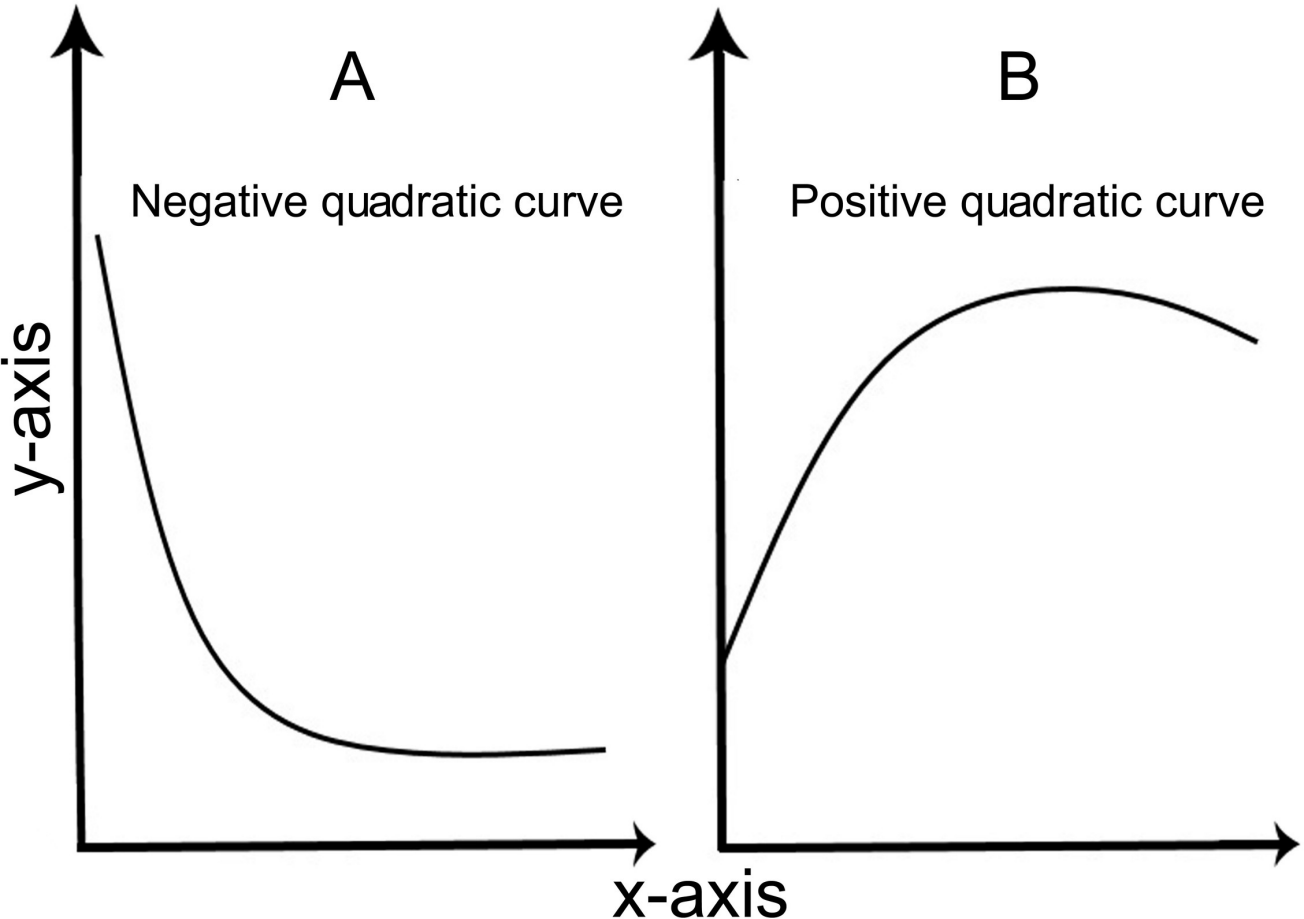
(A) Negative quadratic curve characterizing decay of products through the Arrhenius model and (B) positive quadratic curves for cucurbitacin-containing phytonematicides.

The widely used Arrhenius model for establishing shelf-life decay in food products had been dependent on fitting data of lead chemical compounds to n^0^, n^1^, n^2,^ and n^x^ reaction orders [1–4]. Primary lead chemical compounds in food produce or products include mineral malnutrition elements and a wide range of micronutrient malnutrition substances [5, 6]. In pesticides, lead chemical compounds are technically referred to as active ingredients, with Nemarioc-AL and Nemafric-BL phytonematicides being cucurbitacin A (C_32_H_48_O_9_) and cucurbitacin B (C_32_H_48_O_8_), respectively [7]. The two products, produced from two different *Cucumis* species, with centers of biodiversity in South Africa [8], had since attained cosmopolitan status as invasive plant species [9]. Conventional synthetic chemical nematicides had been withdrawn from the agrochemical markets due to their widespread drawbacks, which included environment-unfriendly and sustainability issues [8]. The withdrawal created opportunities for sustainable alternatives such as phytonematicides [10], with pervasive knowledge gaps [7], including but not limited to information on shelf-life decays of the new products [2, 3]. Since the withdrawal of fumigant nematicides, cucurbitacin-containing phytonematicides had been at the forefront of alternative management options for nematodes [7]. The products consistently suppressed population densities of plant-parasitic nematodes on various crops [7, 8, 10]. However, the products have peculiar reaction orders that did not adhere to the Arrhenius model, which is widely used in assessing shelf-life losses in food products [4]. In products compliant to the Arrhenius and the non-compliant cucurbitacin-phytonematicides have the reaction orders characterized by negative (Fig 1A) and positive (Fig 1B) quadratic relations, respectively [1, 7].

For the two phytonematicides to retain their peculiar features, pieces of mature fruits should be oven-dried at 52°C for 72 h, followed by grinding in a Wiley Mill Model 4 (Thomas Scientific, Swedesboro, New Jersey, USA) to increase the surface area during fermentation [7]. In contrast, most plant organs for medicinal purposes are dried at 30 to 50°C to preserve active ingredients from thermal degradation [11]. However, when *Cucumis* fruits were dried at 40°C, the materials were invariably contaminated with *Penicillium simplicissimum* [12]. During drying fruits from the two *Cucumis* species from 52 to 100°C, cucurbitacin A (cuA) declined from 2.8 to 0.25 μg.ml^-1^ and cucurbitacin B (cuB) from 6.8 to 1.0 μg.ml^-1^ [13]. Relative to oven-drying at 52°C, freeze-drying (–18°C and 100% RH) reduced cuA and cuB by 44 and 60%, respectively [13]. As a compromise to manage *P*. *simplicissimum*, fruits were dried at 52°C, which provided moderate and high cuA and cuB concentrations, respectively [14].

Disparities between Nemarioc-AL and Nemafric-BL phytonematicides were associated with differences in their respective active ingredients [7]. CuA is slightly polar with limited solubility in water, whereas cuB is nonpolar and insoluble [15]. Generally, cuA is unstable and decays into cucumin (C_36_H_46_O_9_) and leptodermin (C_36_H_46_O_8_) chemical compounds, which have insecticidal properties [16], but with scant empirically-based information on nematicidal properties. In contrast, cuB with its diverse pharmacological uses [12, 17], is naturally stable [15]. Storage of phytonematicide inventories in open or airtight containers for six months, or the phytonematicides under similar conditions, resulted in cuA versus storage time exhibiting positive quadratic relations, whereas cuB and storage time had positive linear relations [14]. In other words, at six months cuA concentration had already straddled the three phases of density-dependent growth (DDG) patterns [7], whereas cuB concentration was still increasing linearly in the stimulation phase. Consequently, when *Cucumis* fruits were dried at 52°C, gradual changes in cucurbitacin concentrations over storage time failed to comply with the descending reaction orders of the Arrhenius model [1, 4].

Guidelines for estimating shelf-life losses encompass specification of storage conditions, generalized as fixed and unfixed conditions. Fixed conditions are either frozen (–18°C at 100% RH), chilled (5°C at 95–98% RH), temperate (25°C at 75% RH) or tropical (38°C at 90% RH) conditions, whereas unfixed conditions constitute room temperature conditions [18]. Almost always, Arrhenius model reaction orders are established under fixed conditions [1, 4]. Due to the non-conformity of cucurbitacin to Arrhenius model reaction orders, attempts to develop the shelf-life losses for cucurbitacin-containing phytonematicides using the model were not successful. The objective of this study was to develop an alternative model for establishing the shelf-life damages of cucurbitacin-containing phytonematicides prepared from fruits dried at either 52°C and 100°C under fixed chilled and tropical conditions.

## Materials and methods

### Preparation of phytonematicides

*Cucumis myriocarpus* and *C*. *africanus* plants were cultivated in separate fields, with mature fruits harvested at 92 days after transplanting [19] Fruits were washed using chlorine-free tapwater, cut into pieces and dried at 52°C for 72 h. Separate sets of fruit pieces for each *Cucumis* species were dried at 100°C for 72 h to observe if high temperatures would be detrimental to the product as found previously under unfixed conditions [13]. Dried materials were separately ground in a Wiley Mill Model 4 to pass through a 1-mm-pore sieve. Approximately 80 g *C*. *myriocarpus* and 40 g *C*. *africanus* ground materials were separately fermented in hermetically-sealed 20-L-plastic containers (4 × containers) using effective microorganisms (EM) at 30°C for 14 days until pH was at 3.7 [20]. The EM used in the study comprised yeast, photosynthetic bacteria, lactic acid bacteria, actinomycetes and fermenting fungi [7]. Post-fermentation, 200 ml samples were pipetted into 300 ml black plastic containers, which were hermetically sealed.

### Experimental design

The four parallel trials comprised Nemarioc-AL and Nemafric-BL phytonematicides, each in 50 ml brown plastic containers with the phytonematicide manufactured using fruits dried at 52°C (n = 60) and 100°C (n = 60) and conducted under fixed tropical conditions [18]. Another four similar parallel trials were conducted under fixed chilled conditions [18]. Treatments were initiated at 20 days after drying and comprised 0 (at 14-day post-fermentation), 3, 9, 27, 81 and 243 weeks, arranged in a completely randomized design, with ten replications.

### Extracting and quantifying cucurbitacins

At each sampling time, a 1 ml sample per sample was collected once and pH of contents measured from the remaining solutions using a pH meter. Samples were centrifuged at 4,500 rpm (Multi Pro FC5816/FC5816R) for 10 minutes and filtered through a 0.22 μm-pore filter (Miller, Sigma). Cucurbitacins from other chemical compounds were separated using Shimadzu HPLC with isocratic elution S detected with CTO-20A diode array [21]. Cucurbitacins were then sequentially extracted in a wide pore reverse phase C18 (25 cm × 4.0 mm, 5 μm) discovery (Sigma-Aldrich) using 2:3 (v/v) methanol and deionized water as a mobile phase at a flow rate of 1.0 ml.min^-1^ in an oven at 35°C, with UV-absorbance being at 230 nm and monitored for 43 minutes. Pure (98%) cuA and cuB standards (Wuhan ChemFaces Biochemical, Wuhan: China) were each dissolved in methanol and prepared in serial dilutions ranging from 0.00, 0.02, 0.04, 0.06, 0.08 to 1.0 μg.ml^-1^. Retention times and peak areas for subsamples and pure standards were compared.

### Statistical analysis of data

Prior to subjecting the data to analysis of variance (ANOVA), time-frames (0, 3, 9, 27, 81 and 243 weeks) were expressed as exponentials (3^0^, 3^1^, 3^2^, 3^3^, 3^4^ and 3^5^) and then log-transformed using log_3_3^x^ = x log_3_3 = x to homogenise the x-axis intervals [22]. Using concentrations with geometric series and subsequent log-transformation accorded the dataset a normal distribution, which is an important feature in assumptions for subjecting data to ANOVA. In contrast, failure to normalize the data resulted in generated quadratic curves with skewness to the right [22]. Data with normal distribution within the range of the current time-frame accorded parabolic curves with positive quadratic relations that had the descriptive quadratic constants a, b, and c [22]. The parabolic curves generated using Microsoft Excel 2016 were superimposed on Photoshop M.E. to improve the quality of the curves. Unless otherwise stated, treatment effects were discussed at the probability level of 5%.

## Results

### Influence of drying and storage conditions

**Fig 2:**
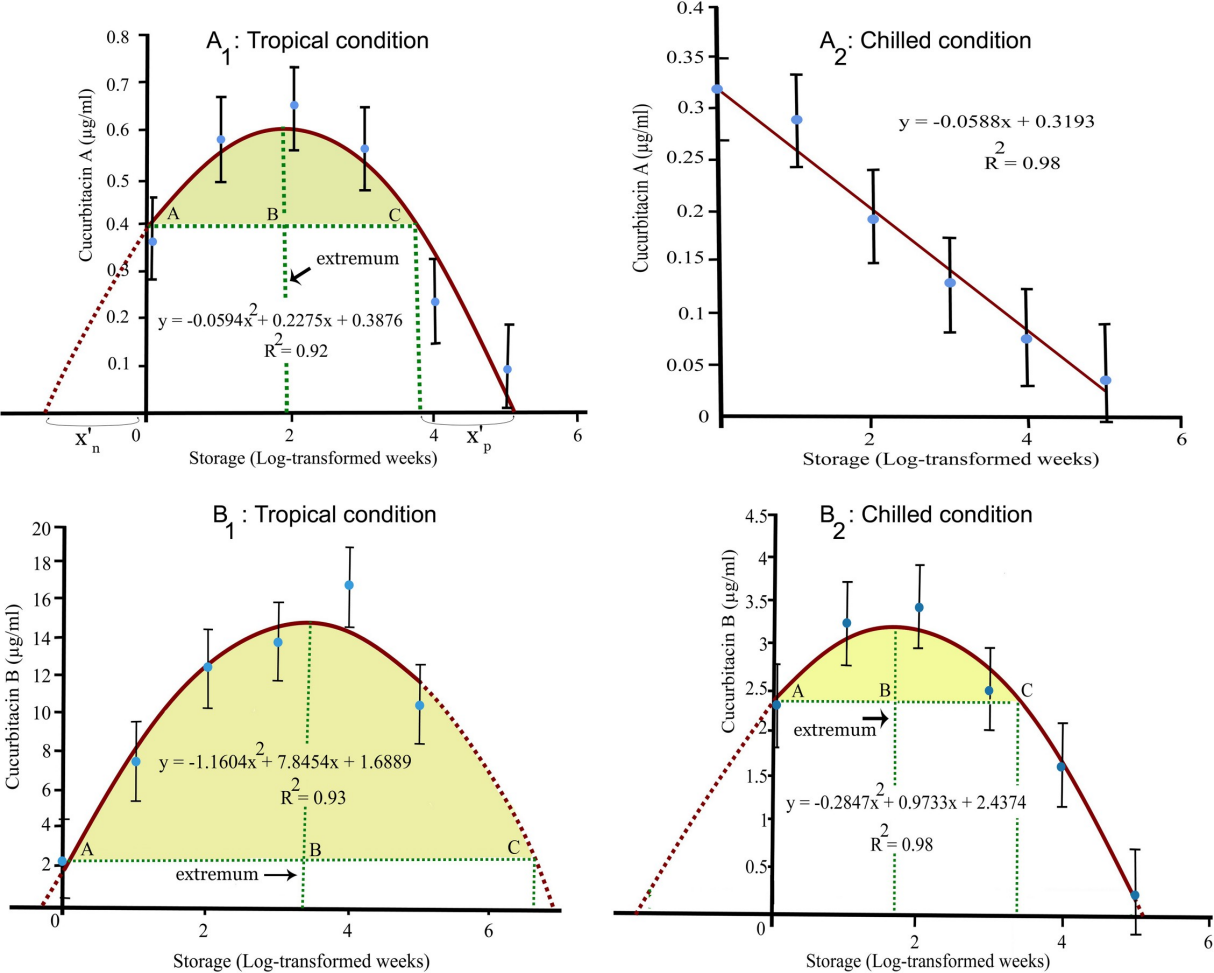
Quadratic models for cucurbitacins in phytonematicides stored under tropical and chilled conditions: Cucurbitacin A and B under tropical (A_1_, B_1_) and chilled (A_2_, B_2_) conditions, with dotted lines on parabolic curves to enhance symmetry, vertical line being the extremum (½ parabola), dotted horizontal line parallel to axis representing shelf-life of cucurbitacin-phytonematicide.

Treatments did not have significant (p ≤ 0.327) effects on cucurbitacins from fruits dried at 100°C under both fixed storage conditions (data not shown). In contrast to fruits dried at 100°C, storage time on cuA and cuB from fruits dried at 52°C was highly significant (p ≤ 0.0021), under tropical conditions contributing 89 and 95% in total treatment variation (TTV) of the respective variables. Those under chilled conditions contributed 94 and 93% in TTV of the respective variables. Under tropical and chilled conditions, cuA versus storage time exhibited positive quadratic (Fig 2A_1_) and negative linear (Fig 2A_2_) relations, respectively, with the models being explained by 92 and 98%, respectively. Under both tropical and chilled conditions, CuB versus storage time also exhibited positive quadratic relations, with the models being explained by 93 and 98% (Fig 2B_1_, Fig 2B_2_).

### Deriving shelf-life from positive quadratic equations

The shelf-life of cucurbitacin-containing phytonematicides could be derived using the precepts of quadratic equation (a*x*^2^ + b*x* + c = 0) [23]. Using information in Fig 2A_1_ as an example, its parabolic curve was extrapolated to intersect the x-axis through dotted lines at the extremes of each curve. The extremum (x = –b/2a) was calculated and expressed as a dotted vertical line that divided the parabola into symmetrical portions. A dotted line parallel to the x-axis from the c-intercept at point A (*x*_1_, y_1_) was drawn, where *x*_1_ = 0 and y_1_ = c = 0.3876 to intersect E at 90° as point B (*x*_2_, y_2_), where *x*_2_ = extremum and y_2_ = y_1_ = 0.3876. The line was then further extrapolated to intersect the parabolic curve at point C (*x*_3_, y_3_), where y_3_ = y_2_ = y_1_ = 0.3876 since line AC was horizontal to the *x*-axis. Also, in other curves, the values of *x*_1_ and *x*_2_ were known, whereas those of *x*_3_ was unknown. In the current study, the value of *x*_3_ depicts the shelf-life of the test phytonematicide. In the quadratic formula (–0.0594*x*^2^ + 0.2275*x* + 0.3875 = 0) for Fig 2A_1_, the extremum would be expressed as [23]:

$$x_{2}=\frac{-b}{2a},$$ Eq 1

Where the constant a is a non-zero digit as expounded in quadratic relation precepts [23]. In each of the three parabolic curves (Fig 2A_1_, 2B_1_ and 2B_2_), to the left of the extrema is a dotted line perpendicular to the x-axis, with the y-axis forming a similar vertical barrier to the right. The distance from any of this vertical lines to the point where the parabolic curve intercepts the *x*-axis is, to enhance clarity in this study, prime *x* (*x*'), with distance to the left being prime *x* positive (*x*'_p_) and that to the right being prime x negative (*x*'_n_), where *x*'_p =_ *x*'_n_. Any point where the parabolic curve intercepts the *x*-axis is a root [23], with each of the parabolic curves in Fig 2 having two roots. Using the quadratic formula (*a*x^2^ + *b*x + c = 0) when c ≠ 0, roots *x*'_p_ and *x*'_n_ in Fig 2A_1_, 2B_1_ and 2B_2_ could be derived from:

$$x=\frac{-b\pm \sqrt{b^{2}-4ac}}{2a}$$ Eq 2

Given that the shelf-life of the test phytonematicides is line AC, all coordinates of points in Fig 2 could be derived from Eqs 1 and 2, except for *x*_3_ for point C. In Fig 2A_1,_ for example, since *x*'_p_ = *x*'_n_, subtracting the absolute value of *x*'_n_ from the positive root (*x*_p_) would result in *x*_3_, the *x*-coordinate for line AC, which represents the shelf-life (S1 Table). However, since the time-frame was log-transformed to normalize the data, antilogarithms should be calculated to record the shelf-life in real weeks (S1 Table).

## Discussion

The negligent cucurbitacin concentration confirmed previous observations where cuA and cuB for fruits dried at 100°C were the lowest [13]. Cucurbitacins are biosynthesized through 1-deoxy-D-xylulose 5-phosphate/2-C-methyl-D-erythritol 4-phosphate (DOXP/MEP) pathway, which alternates with the classical mevalonate pathway in the mitochondria [24]. The DOXP/MEP pathway biosynthesizes carotenoids, phytols, isoprenes, mo-, di-, tetraterpenes and plastoquinones, whereas the mevalonate pathway the sterols, sesquiterpenes and triterpenes [24, 25], with the latter being central in the biosynthesis of cucurbitacins. Generally, the isolated isoprenoids in the alternate pathways are high-energy molecules [24], which become stable once the final product is biosynthesized [25]. In *C*. *myriocarpus* plants, several isoprenoid-precursors are translocated from other organs to roots and fruit [7], where they are eventually compartmentalized as cuA, which decays rapidly, with cucumin and leptodermin being biosynthesized [15]. In contrast, in *C*. *africanus*, cuB is biosynthesized as expounded and stored in all plant organs, with bias being towards accumulation in fruits [26]. The biosynthetic rates are limited by the availability of appropriate isoprenoid-precursors and enzymes [23]. Bottlenecks in biosynthetic pathways are often induced when factors such as temperature, pH and microbial degradation interfere with isoprenoid-precursors and enzymes [17, 23]. At least 18 enzymes required to drive biosynthetic pathways of cucurbitacin E and I had been identified [25]. The observations [25] provided some physiological clarity on the peculiar behavior of cucurbitacins when mature fruits were dried at 52°C as opposed to 100°C. Generally, cuA and cuB are thermostable, with their sea-level boiling points being at 731 and 699°C, respectively [13].

Under tropical conditions, shelf-life loss of Nemarioc-AL phytonematicide occurred at 35 weeks, and under chilled conditions, the product had limited shelf-life, supporting the view that cuA was highly unstable [15]. The unstableness of this active ingredient, as confirmed in the current study, was temperature-dependent [14]. Also, the dependence supports the view that cucurbitacin-containing phytonematicides behave differently to the precepts of the Arrhenius model [1]. In the current study, it was shown that the peculiar behavior occurs only when fruit pieces from *Cucumis* species were dried at 52°C, which is close to the temperature used in drying plant materials for medicinal purposes [11]. In Nemarioc-AL phytonematicide, freezing winter temperatures in the context of the expected extremes in climate change could interfere with the efficacy of the product on nematode suppression under open-field cropping systems.

In contrast, during hot summer months, the product would be suitably positioned to manage nematode population densities in the rhizosphere. The benzyl groups in cucumin and leptodermin chemical compounds, were previously shown to have potent insecticidal properties [16], with limited information on their nematicidal properties. The characteristic decline in cuA under both conditions could suggest that the isoprenoid-precursors that were already in fruit at drying continued along the DOXP/MEP and mevalonate biosynthetic pathways to form cuA. However, the latter undergoes degradation activities to produce cucumin and leptodermin. Essentially, it is essential to note that degradation of CuA to the two products is not a reductionist process, but increases the biosynthesis and accumulation of cucumin and leptodermin, which have increased carbon, oxygen and hydrogen atoms.

Under fixed tropical and chilled conditions, shelf-life losses for Nemafric-BL phytonematicide were distinctly different from those of Nemarioc-AL phytonematicide under tropical conditions. The shelf-life period for the product under chilled conditions was similar to that of Nemarioc-AL phytonematicide under tropical conditions. The extended shelf-life of 825 weeks under tropical conditions for Nemafric-BL phytonematicide appears to surpass those of most commercially available bio-based products. For instance, Biogard, with active ingredient *Ampelomyces quisqualis*, has the shelf-life of 192 weeks at 4°C [27]. The extended shelf-life of Nemafric-BL phytonematicide also supported the view that temperature has a strong influence on the biosynthetic abilities of cucurbitacins [13]. Relative to fixed chilled conditions (35 weeks), fixed tropical conditions increased the shelf-life of Nemafric-BL phytonematicide (825 weeks) by 2257%. Fixed tropical conditions under which shelf-life losses were delayed by going through the phases of DDG patterns mimicked conditions for distribution channels and on-farm storage at room temperatures in tropical regions. Both Nemarioc-AL and Nemafric-BL phytonematicides would be user-friendly when intended for use in the tropical areas, where most plant-parasitic nematodes are increasingly becoming economic pests due to climate change [7]. At the end of the shelf-life, cucurbitacin concentrations were equal to those at the end of the fermentation process, that is, y_3_ = y_1_. Obviously, by the end of the established shelf-life, as reiterated in other shelf-life models [1–4], it does not imply that the product could no longer be used, but the shelf-life cautions that efficacy might no longer be consistent with the claims on label instructions. During such end times, mainly when disposal activities are contemplated, the products should still be handled with great caution to avoid environmental contamination and health-hazards to non-target animals.

Although temperature arguably played essential roles in behavioral patterns of cuA and cuB, the factor could unlikely have been the lone factor responsible for shelf-life losses in cucurbitacin-containing phytonematicides. In other shelf-life studies [28–30], degradation of active ingredients was implicated as the primary factor that increased the shelf-life losses, with the focus being on potential degradation-inducing factors. For instance, cucurbitacin E in bitter Hawkesbury watermelon (*Citrullus vulgaris* Thunb.) was largely compromised by both microbial and low pH degradation [28–30]. A persistent decline in pH could deactivate most enzymes, thereby disrupting biosynthetic pathways that rely on enzyme activities to reduce activation energy [28, 29]. In our study, the post-fermentation pH of phytonematicides was at 3.7, with storage time having no significant effects on the variable. Mean pH values by the end of the storage time for Nemarioc-AL and Nemafric-BL phytonematicides were 3.85 and 2.70, respectively. Storage temperature and cucurbitacin type were the primary factors associated with cuA and cuB shelf-life losses, but there could also be contributions from a wide range of secondary factors such as pH or natural decay followed by biosynthesis of alternative chemical compounds [28–30].

## Conclusion

The shelf-life loss equations for cucurbitacin-containing phytonematicides were phytonematicide specific- and storage condition-specific. The equations were derived from empirically-based static tests that generated quadratic equations and their related derivatives. Under both conditions, the shelf-life loss equation was viewed as being twice the extremum value of the density-dependent degradation of the active ingredient of the test phytonematicide over storage time. Under tropical conditions, Nemarioc-AL and Nemafric-BL phytonematicides could be stored up to 35 and 825 weeks, respectively. Under chilled conditions, the model could not predict the shelf-life loss for Nemarioc-AL phytonematicide, whereas that for Nemafric-BL phytonematicide was reduced to 35 weeks. In both phytonematicides, the shelf-life losses were dependent upon temperature used in fruit drying, cucurbitacin type, and storage condition. The derived shelf-life loss equations would contribute to policy- and business-making decisions with regards to the contribution of cucurbitacin-containing phytonematicides to climate-smart agricultural practices.

## Supporting information

S1 Data The shelf-life data file of cucurbitacin-containing phytonematicides over time. (XLS)

S1 Table Shelf-life (*x*_3_) of cucurbitacin-containing Nemarioc-AL and Nemafric-BL phytonematicides in real weeks. (DOCX)
