# Supplementary material for: Shelf-life in cucurbitacin-containing phytonematicides: Non-conformity to Arrhenius model
Source: PLoS One. 2020 Feb 12;15(2):e0227959. doi: 10.1371/journal.pone.0227959 (PMC7015409; doi:10.1371/journal.pone.0227959)
Supplement: S1 Table — (DOCX) [file pone.0227959.s002.docx]

**S1 Table.** Shelf-life (*x*_3_) of cucurbitacin-containing Nemarioc-AL and Nemafric-BL phytonematicides in real weeks.

| **Product** | **Condition** | **Quadratic equation for**  **computing roots** | **Roots** | | **Shelf-life (*x*_3_)^z^** | |
| --- | --- | --- | --- | --- | --- | --- |
|  |  |  | ***x*_p_** | ***x*_n_** | **log wk** | **Real wk** |
| Nemarioc-AL | Tropical | –0.0594*x*^2^ + 0.2275*x* + 0.3876 = 0 | 5.14 | –1.28 | 3.86 | 35 |
| Nemafric-BL | Tropical | –01.1604*x*^2^ + 7.8454*x* + 1.6889 = 0 | 7.00 | –0.21 | 6.79 | 825 |
| Nemafric-BL | Chilled | –0.2847*x*^2^ + 0.9733*x* + 2.4374 = 0 | 5.14 | –1.28 | 3.86 | 35 |

^z^Log-transformed week and real week, where log weak = *x*_p_ – **|***x*_n_**|**, where **|***x*_n_**|** is the absolute value.
